# A Dangerous Mimic: Chronic Pancreatitis Masquerading As Pancreatic Adenocarcinoma

**DOI:** 10.7759/cureus.19795

**Published:** 2021-11-21

**Authors:** Zoe Li, Irim Salik

**Affiliations:** 1 Department of Anesthesiology, Westchester Medical Center, Valhalla, USA

**Keywords:** pediatric cancer, pediatric surgery, whipple procedure, chronic pancreatitis, pancreatic adenocarcinoma

## Abstract

Pancreatic adenocarcinoma (PAC) is the most common form of pancreatic cancer in adults, although extremely rare before the age of 40 years. It is known that the cytology of chronic pancreatitis can mimic pancreatic adenocarcinoma. We present a case of a 13-year-old male with chronic pancreatitis that was misdiagnosed as PAC. The patient subsequently underwent a Whipple procedure, highlighting the importance of a correct diagnosis prior to undergoing invasive surgical procedures.

## Introduction

It has been commonly reported in the literature that chronic pancreatitis (CP) can mimic pancreatic adenocarcinoma (PAC), leading to avoidable surgical intervention and increased postoperative morbidity. Pancreatic adenocarcinoma (PAC) is the fourth leading cause of cancer mortality in the United States with an overall five-year mean survival rate of 10% [1]. There is a lack of standardized guidelines in assessing potential pancreatic cancer masses [2]. The diagnosis typically involves clinical suspicion, followed by imaging studies including computed tomography (CT) and/or MRI, and lastly confirmation by fine-needle aspiration (FNA) or endoscopic ultrasound (EUS) [2]. Although the Whipple procedure improves survival for patients with localized disease, the morbidity of PAC can be as high as 50%, which makes proper identification of benign disease paramount [3]. While the anesthesiologist plays a pivotal role during surgical resection of the mass, a multidisciplinary team including a radiologist, pathologist, general surgeon, and the oncologist play roles in the appropriate and timely diagnosis of PAC. We present the case of a teenager who was inappropriately diagnosed with PAC based on FNA biopsy and underwent a Whipple procedure. 

## Case presentation

**Figure 1:**
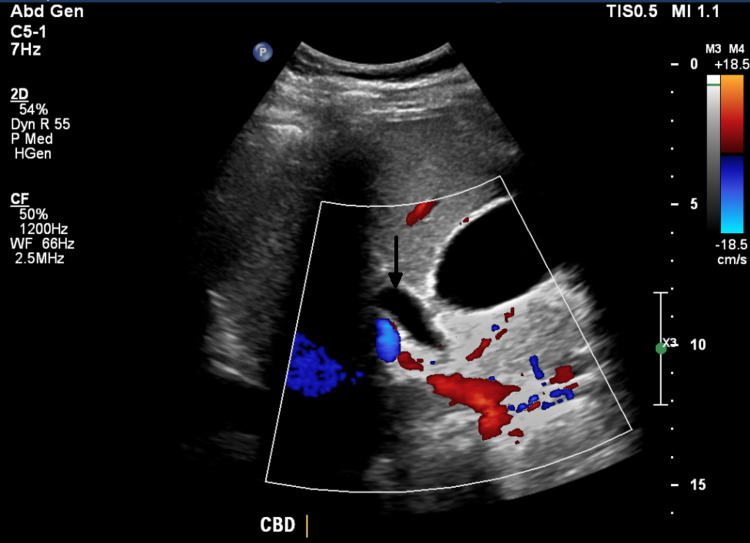
Ultrasound of common bile duct. Dilation of common bile duct to 0.9 cm. Arrow shows dilation of the common bile duct to 0.9 cm as it abuts the gallbladder. CBD: common bile duct.

**Figure 2:**
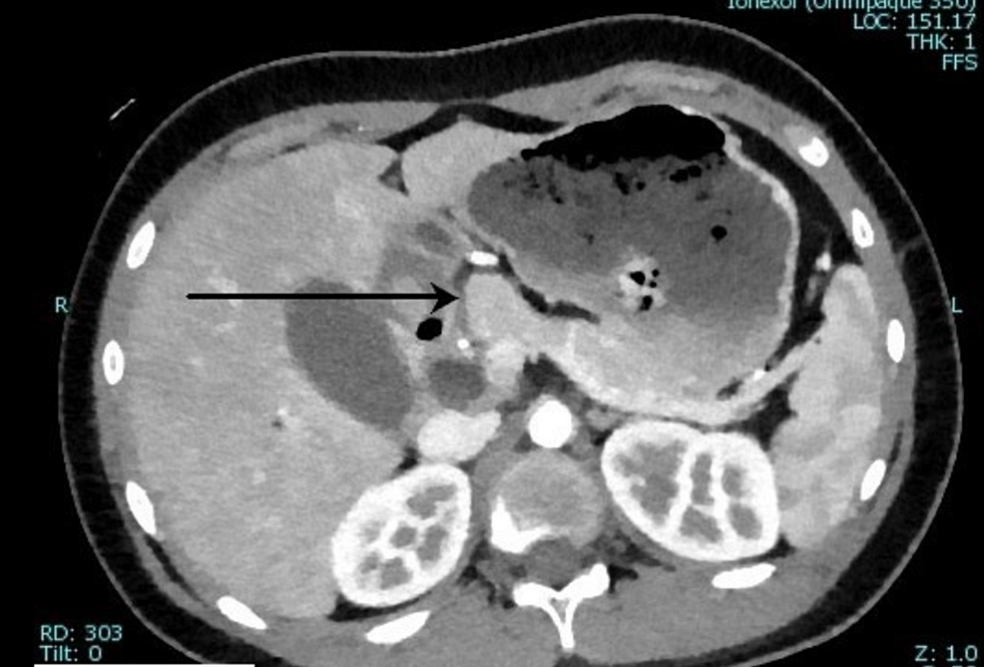
Pancreatic head mass on CT. Arrow shows a pancreatic head mass measuring 2.8 × 2 × 3 cm. The mass is ill-defined and mildly hypoenhancing on the arterial phase. CT: computed tomography.

A 13-year-old male presented with abdominal pain, a history of Helicobacter pylori infection six months prior, and abnormal liver function tests (aspartate aminotransferase 518 U/mL, alanine transaminase 779 U/mL, alkaline phosphatase 479 U/L, and gamma-glutamyl transferase 395 U/L). The patient’s carbohydrate antigen (CA 19-9) (19.3 U/mL) and carcinoembryonic antigen (CEA) (0.6 ng/mL) were within normal limits. No family history of pancreatic cancer was noted. Abdominal ultrasound revealed a dilated common bile duct (0.9 cm) and a computed tomography (CT) scan demonstrated a pancreatic head mass (see Figures 1, 2). FNA from the pancreatic mass revealed adenocarcinoma. The patient presented for a Whipple procedure and was subsequently induced and intubated atraumatically with a 6.5 cuffed endotracheal tube. A left radial arterial line and a 5 French double lumen central venous catheter were placed. A thoracic epidural was placed at T9-T10 and a ropivacaine infusion was run at 3 mL/hr intraoperatively. A successful pancreaticoduodenectomy was performed and the patient was extubated postoperatively and admitted to the intensive care unit. He was transferred to the floor on postoperative day (POD) 2 and the epidural was removed on POD 4. The patient was discharged on POD 13 with a pancreatic drain in place. The subsequent pathology of the pancreatic tissue specimen obtained intraoperatively was classified as non-malignant, revealing localized pancreatitis with both chronic and acute inflammation.

 

## Discussion

Our patient was mistakenly diagnosed with pancreatic adenocarcinoma (PAC) on the basis of FNA of the pancreatic head mass. The incidence of pediatric PAC is very rare, with a median age of diagnosis at 70 years of age [1]. Younger patients generally have an associated genetic syndrome, including ataxia-telangiectasia, hereditary pancreatitis, hereditary non-polyposis colon carcinoma, and Puetz-Jeghers syndrome. It is difficult to ascertain the incidence of pediatric PAC because of its rarity. In patients under 40 years of age, its incidence was found to be approximately 0.3% in patients <40 years, and 0.1 % in patients <20 years [4]. Differential diagnosis of pediatric PAC includes other more common neoplasms such as pancreatoblastoma (most common pediatric pancreatic tumor), solid-pseudopapillary tumor, and islet cell tumors [3]. Upon postoperative review of specimen pathology, the patient was diagnosed to have idiopathic fibrosing pancreatitis, a rare form of chronic pancreatitis, based on the clinical history and histomorphology.

There are a number of etiologies for chronic pancreatitis, including alcoholism, malignancy, trauma, metabolic derangements, choledocholithiasis, and hereditary disorders. Management is usually conservative, including parenteral nutrition, bed rest, and alcohol and smoking cessation. Surgical intervention is undertaken, in cases of gastric outlet or biliary obstruction caused by chronic strictures, fibrosis, and duodenal wall thickening. A Whipple procedure is indicated in CP if a patient suffers from intractable pain, weight loss, pancreatic insufficiency, or a pyloric structure [5].

Of great concern, multiple studies as summarized by Farrell have shown that the accuracy rate for differentiating PAC from chronic pancreatitis with EUS ranges from 63% to 76% [6]. An erroneous diagnosis can be due to the presence of a focal hypoechoic mass, vascular involvement, or local mass extension to surrounding structures [6]. It is challenging to differentiate PAC from chronic pancreatitis (CP) based on cytology, as both exhibit large nuclei and degenerative vacuoles [7]. In PAC, cellular features include epithelial cell neoplasm with glandular differentiation, which can range from infiltrating single cells with mucin production to solid sheets of neoplastic cells. Stains such as periodic acid-Schiff (PAS) and mucicarmine as well as immunohistochemical labeling can help in the evaluation of suspected infiltrating adenocarcinoma. Most PACs express cytokeratins 7, 8, 13, 18, and 19, as well as more common markers such as CEA and CA 19-9 [8]. In this patient, Diff-Quik (DQ) and Papanicolaou (PAP) stains were performed on the four smears prepared from the FNA of the pancreatic mass.

Inflammatory conditions that mimic PAC include paraduodenal pancreatitis (PDP) or “groove pancreatitis,” autoimmune pancreatitis (AIP), mass forming chronic pancreatitis, obstructive chronic pancreatitis, intrapancreatic splenules, and intraductal papillary mucinous neoplasm. A disease characterized by recurrent inflammation leading to gland atrophy, CP causes fibrosis and pancreatic duct strictures and dilatation. In some cases, CP can lead to a mass-like inflammatory lesion in the pancreatic head. Kennedy et al. estimate that between 5% and 35% of patients who undergo a Whipple procedure actually have a non-neoplastic inflammatory mass [9].

Although imaging alone may not provide an adequate distinction, there are a number of techniques available to differentiate between PAC and CP. The duct-penetrating sign first described by Ichikawa et al. on magnetic resonance cholangiopancreatography (MRCP) strongly favors an inflammatory etiology for a pancreatic mass [10]. Cases of AIP are highly responsive to steroids, and patients can be trialed this way prior to surgical intervention. In up to 95% of patients, IgG4 serum levels are increased [11]. In younger patients, there is some predilection for AIP type 2, characterized by granulocytic epithelial lesions (GELs), and an association with Crohn’s disease or ulcerative colitis [11]. An ultrasound or CT that reveals a non-dilated pancreatic duct favors a diagnosis of CP as opposed to cancer. AIP is also the likely diagnosis if a CT scan reveals a diffusely enlarged pancreas with a capsule-like rim, or if other organs are involved including the kidneys, salivary glands, or common bile duct. Factors indicating PAC include a low-density mass, cut off of the pancreatic duct, atrophy of the distal pancreas, and distant metastases. In this patient, the IgG4 stain was performed on the cells obtained from the surgical specimen, revealing 80% positive IgG4 stain per high power field (HPF). However, despite fibrosis and high IgG4 percentage, AIP was seen as less likely because of the scant amount of plasma cells and lymphoid infiltrate.

Further complicating matters, Chari et al. concluded that in 30% of cases, AIP cannot be distinguished from PAC without a core biopsy, surgical resection, or a steroid trial [12]. The role of pancreatic biopsy is also somewhat controversial, as Detlefsen et al. utilized core pancreatic biopsies to diagnose AIP using six microscopic features, including GELs, IgG4-positive per HPF, eosinophilic granulocytes per HPF, fibrosis with inflammation, venulitis, and lymphoplasmacytic infiltration [13]. Even with the six aforementioned criteria, AIP was detected in only 86% of cases [12]. In our patient, IgG4 measurements were taken after the Whipple was performed and there was no adequate imaging of the pancreatic duct, both required for diagnosis based on the histology, imaging, serology, other organ involvement, and response to steroid therapy (HISORt) criteria defined by the Mayo Clinic [14]. In addition, the normal level of CA 19-9 was not given enough credence in this patient, as levels of >300 U/mL are considered to be pathognomonic for the disease.

## Conclusions

Overall, it was found that even with a diagnosis of PAC on FNA, further testing should be undertaken prior to surgical excision, specifically in a pediatric patient in which carcinoma is rare. There should be a definitive diagnosis before subjecting a patient to lifelong Whipple physiology, which can include gastric dysmotility, pancreatic insufficiency, diabetes, and nutritional deficiencies. We have now changed the standard of care at our institution so that in pediatric patients, a core biopsy, and/or steroid trial is mandatory prior to a pancreaticoduodenectomy.
